# Mind wandering is not always harmful in sports: the role of its content

**DOI:** 10.3389/fpsyg.2024.1348893

**Published:** 2024-08-07

**Authors:** Jieling Li, Chuangye Li, Shuangpeng Xue, Yuxiu He

**Affiliations:** ^1^Physical Education Postdoctoral Research Station, Hebei Normal University, Shijiazhuang, China; ^2^School of Physical Education, Hebei Normal University, Shijiazhuang, China; ^3^Key Laboratory of Measurement and Evaluation in Exercise Bioinformation of Hebei Province, Shijiazhuang, China

**Keywords:** athlete, content, frequency, mind wandering, performance

## Abstract

**Objective:**

Mind wandering (MW) among athletes during training and competition can lead to poor performance. However, MW has also been found to have positive effects. This study aims to clarify the causes of the bidirectional (negative and positive) effects of MW in the sports context, specifically focusing on whether these effects are related to the content of MW.

**Methods:**

A total of 846 Chinese athletes completed the Chinese version of the MW scales. The survey data were tested for common method biases. Subsequently, Pearson correlation analysis and structural equation modeling were performed using SPSS 25.0 and Mplus 7.0.

**Results:**

The frequency of MW can positively predict its bidirectional effects. MW content plays an important role in the relationships between MW frequency and its negative and positive effects, but the direction of influence varies depending on the content.

**Conclusion:**

MW in sports is not always harmful, and its content plays an important role. These findings suggest that managing MW content may be a promising MW intervention method for improving performance in sports.

## Introduction

Mind wandering (MW) refers to an experience in which the mind shifts away from the present task to unrelated inner thoughts (Smallwood and Schooler, 2006). It is a common phenomenon among human beings, accounting for 30–50% of people's waking hours (Kane et al., 2007). For athletes, the term MW refers to the mind not being tied to sporting tasks but instead focused on internal thoughts. Athletes often experience MW while on the train or in competitions. For example, tennis player Roger Federer said, “In a match, my mind wanders at times, sometimes a song comes to my mind, sometimes you are thinking about what's going to happen tomorrow, what you're going to do tonight, you might think about anything” (Netease Sports News, 2011).^1^ In another example, during the Tokyo 2020 Olympics, Ukraine's Serhiy Kulish shot his rifle into another competitor's target. After the match, he recalled that his clothes felt slightly uncomfortable during the final moments of the match, causing his mind to wander (Sohu, 2021).^2^ The characteristics of MW are multidimensional, including whether it is voluntary (Seli et al., 2015, 2016), temporal directionality (Smallwood et al., 2009), or meta-awareness (Smallwood et al., 2008). There are many theories explaining how and why MW occurs. Among them, the “decoupling hypothesis” explained in terms of cognitive resource allocation suggests that MW arises due to the coupling of attention to internal processing while decoupling it from task-relevant information (Smallwood and Schooler, 2006; Smallwood, 2010). Based on the “decoupling hypothesis,” the athlete experiencing MW consumes cognitive resources.

However, the effect of MW is not homogeneous. MW has negative and positive effects (Mooneyham and Schooler, 2013; Torres-Irribarra et al., 2019; Salavera and Usán, 2020). Regarding the negative effects, MW can lead to dangerous driving behavior (Burdett et al., 2016; Albert et al., 2018) and cause nautical observers to miss critical radar signals (David et al., 2015). The sports field is no exception. MW has been confirmed to be very common among athletes during sporting events (Miś and Kowalczyk, 2021), and it can produce a series of negative effects, such as detrimental distraction (Latinjak, 2018), motor errors, energy consumption, emotional fluctuation, injuries, decreased training effectiveness, and poor competitive performance (Li and Yao, 2016). Regarding the positive effects, MW can anticipate and plan personal future goals (Baird et al., 2011). MW is also related to creativity, patience, cognitive control (Smallwood and Andrews-Hanna, 2013), and new solutions to old problems (Baird et al., 2012). Unexpectedly, the positive effect of MW has also been observed in the field of sports. For example, MW was found to be related to helpful distraction and sudden insight (Latinjak, 2018). MW during training for long-distance running enhances an individual's mood (Miś and Kowalczyk, 2021). Interviews with athletes have also revealed that MW is not always harmful; it also exhibits beneficial functions, such as improving one's mood and reducing fatigue (Li and Yao, 2016). Although the study of MW in sports is still in its infancy, the coexistence of its advantages and disadvantages has been demonstrated in the above studies. Therefore, instead of blindly asking athletes to reduce MW, the reason behind its bidirectional (negative and positive) effects should be clarified first.

To date, however, the reason for the bidirectional effects of MW remains unclear. Thought content profiles frequently vary considerably among individuals, and thus, the heterogeneity of MW must be considered. Therefore, it is necessary to consider the content characteristics of MW. Smallwood and Andrews-Hanna (2013) proposed the “content regulation hypothesis,” which suggests that the effects of MW are likely to originate from their different contents. Repetitive thinking of negative and self-related content and excessive focus on memories and future thoughts are associated with negative emotions. However, adaptive, constructive, and highly self-relevant MW contents provide a means to address current concerns and facilitate insightful and creative problem-solving (Smallwood and Andrews-Hanna, 2013). Intra-individual analyses indicated that negatively valenced MW and MW without awareness correlated with worse performance (Welhaf et al., 2024). The “content regulation hypothesis” also states that MW content is time-oriented, and different time orientations (e.g., past and future) will have varying effects. Research has shown that future-pointing MW allows individuals to envision and plan for the future, which is closely related to personal goals.

In contrast, past-pointing MW is associated with negativity and low wellbeing; past-pointing wandering tends to be associated with negative emotions, while future-pointing MW is related to positive emotions (Smallwood and Andrews-Hanna, 2013). In the sports domain, Miś and Kowalczyk study (2021) concluded that future-pointing MW appears to promote positive mood shifts during runners' training. Thus, the content of MW largely determines its effect.

The “context regulation hypothesis,” another theory proposed to analyze the effect of MW, argues that explaining the effect of MW also requires focusing on the task context (Smallwood and Andrews-Hanna, 2013). The training ground and the competition terrain where athletes are located differ from daily life situations. Thus, we should explore MW content corresponding to reality in sports situations. In addition, the “current concerns hypothesis” specifies that an individual's MW content is related to his/her personal goals or unaccomplished tasks (Klinger and Cox, 1987). Therefore, by combining the perspectives of the “content regulation hypothesis,” “context regulation hypothesis,” and “current concerns hypothesis,” the effect of MW on athletes must consider its content features and combine the sports context with the current concerns of athletes.

**Figure 1:**
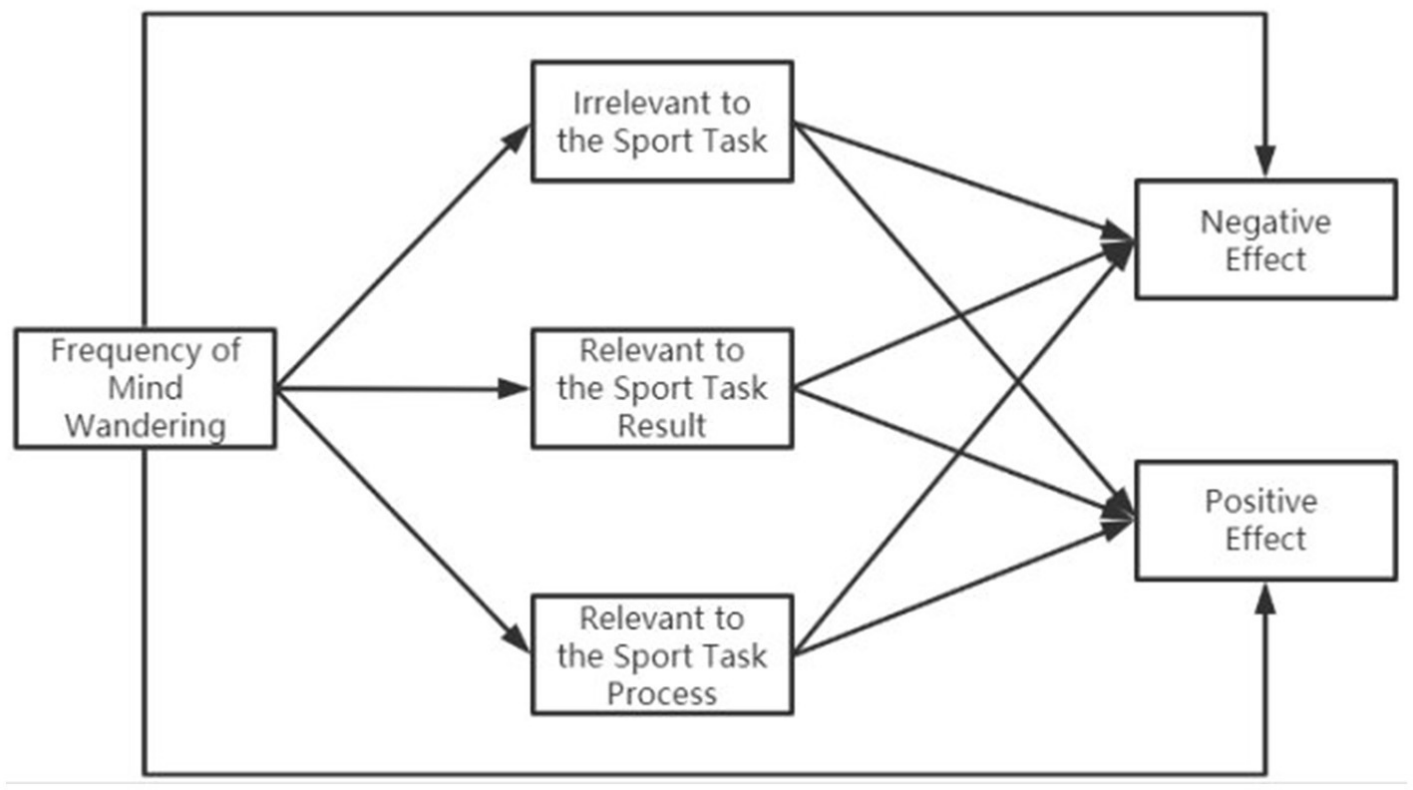
Hypothesis model.

Previous studies have categorized the content of MW considering the characteristics of the sports context and the current concerns of the athlete (Li and Yao, 2016). On this basis, the present study considers the content of athletes' MW and uses structural equation models to explore the bidirectional (negative and positive) effects of MW on training and competition contexts and whether different MW contents play a mediating role. The theoretical path model is shown in Figure 1, and the following hypotheses are proposed: (1) The frequency of athletes' MW can directly predict its positive and negative effects. (2) Different MW contents play different roles in the frequency of MW and negative effects. (3) Different MW contents play different roles in the frequency of MW and positive effects. The results of this study will provide scientific guidance for reducing the negative effect of MW on sports performance and guiding it toward a positive effect.

## Materials and methods

### Participants

The present study was approved by the relevant Ethics Committee (2023LLSC031), and all the subjects who participated in the survey provided informed consent. The participants were from professional sports teams and competitive sports schools in eight cities in China, namely Beijing, Tianjin, Shanghai, Chongqing, Xi'an, Chengdu, Zhengzhou, and Wenzhou. They had to train for more than 10 h a week and had to participate in competitions at or above the provincial level. Questionnaires were distributed online (web-based and password-protected) and on paper. Data were collected from 1,186 participants, and 340 were excluded by setting two polygraph questions and checking for regular responses, achieving an effective recovery rate of 71.332%. The final sample was 846 Chinese athletes. Among them, there were 570 men and 276 women: 357 athletes in the physique-dominated event group and 489 athletes in the skill-dominated event group; 379 athletes at level 2 or above; and 467 athletes below level 2. The average age of the participants was 20.450 years (*SD* = 2.407). They had participated with their respective teams for an average of 5.880 years (*SD* = 3.815).

### Measures

A series of scales developed in a previous study (Li and Yao, 2017) on athletes' MW was selected. The participants responded using a 5-point Likert-type scale from 1 (*never*) to 5 (*always*).

#### Frequency of MW

The frequency of MW among the athletes was measured using the *MW Cause Scale*. This scale investigates which situations make athletes more prone to MW during training and competition. Consequently, the total score measures how frequently MW occurs among athletes. This scale contains five dimensions: weak attentional control (three items; e.g., “When motor difficulty is low, my mind will wander”), spontaneous thinking (six items; e.g., “Near the end of training, my mind cannot help but wonder”), psychological gap (three items; e.g., “I zone out when the organization of the event changes suddenly”), competition mood (four items; e.g., “My mind will wander when I get too nervous”), and somatic sensation (five items; e.g., “My mind wanders when I am tired”), for a total of 21 items. To meet the model fit criteria, one item from the spontaneous thinking dimension was removed, leaving a total of 20 items. The internal consistency reliability for each dimension was 0.661, 0.806, 0.710, 0.800, and 0.826. The scale had good construct validity (χ^2^*/df* = 2.968, TLI = 0.911, AGFI = 0.894, CFI = 0.924, RMSEA = 0.059; Li and Yao, 2017).

#### Content of MW

The different MW contents of the athletes were measured using the *MW content Scale*. This scale is used to investigate the three different types of MW content during training and competition situations: irrelevant to the sports task (three items; e.g., “Remember something delicious that you have eaten before”), relevant to the sports task result (three items; e.g., “Remember the honor of victory”), and relevant to the sports task process (three items; e.g., “The change in the field is the change in the content of my wandering mind”), for a total of nine items. The internal consistency reliability for each dimension was 0.731, 0.756, and 0.723. The scale had good construct validity (χ^2^*/df* = 2.968, TLI = 0.957, AGFI = 0.948, CFI = 0.972, RMSEA = 0.059; Li and Yao, 2017).

#### Effect of MW

The bidirectional effects of the athletes' MW were measured using the *MW Result Scale*, which investigates the effect of MW during training and competition situations. It includes two dimensions: negative effect (seven items; e.g., “My competition scores will go down”) and positive effect (five items; e.g., “Relieve tension during the game”). Negative effects include motor errors, energy consumption, emotional fluctuation, injuries, decreased training effectiveness, and competition performance. Positive effects include decision-making and emotional regulation. The negative dimension has seven items, while the positive dimension has five items, for a total of 12 items. To meet the model fit criteria, two items from the negative dimension and one item from the positive dimension were eliminated, leaving a total of nine items. The internal consistency reliability for each dimension was 0.820 and 0.844. The scale had good construct validity (χ^2^*/df* = 2.969, TLI = 0.941, AGFI = 0.933, CFI = 0.953, and RMSEA = 0.059; Li and Yao, 2017).

### Data analysis

We computed means, standard deviations, and correlations for all the variables by using SPSS 22.0. Confirmatory factor analysis (CFA) was conducted using Mplus 7.0 to examine the reliability, convergent validity, model fit, and multiple mediating effects. Among these, the test for multiple mediating effects yielded comparative mediating effects, allowing the researchers to determine which multiple mediating variables exerted a stronger effect. This study used the bootstrap method for multiple mediating effect analysis. This method involves the process of taking a large bootstrap sample (sample size = 1,000 for this study) and obtaining statistics through repeated sampling with put-backs. This method does not require normality assumptions or large samples, and it is useful for small to medium-sample analyses. Bootstrap methods include the percentile bootstrap method and the bias-corrected percentile bootstrap method. Both are presented in the reporting of the results of this study. When the confidence interval does not contain 0, then the indirect effect is significant.

## Results

### Common method bias test

To verify whether the survey has a significant systematic error, the Harman one-way method was used to test the common method bias of factors (Podsakoff et al., 2003). The analysis results showed that seven factors out of 42 had a characteristic root >1, and the percentage of the first factor explainable was 32.576%, which was lower than 40%, indicating no significant common method bias for the factors used in this study.

### Reliability and validity tests of the scale

As shown in Table 1, compositional reliability and convergent validity were calculated to test the reliability of the questions and the ability of the dimensions to explain the questions. The component reliability of each dimension was close to or above 0.7. AVE refers to the average explanatory ability to determine the dimensions of an item. All the dimensions were >0.36, indicating that they were within an acceptable range. To test discriminant validity among the dimensions, this study calculated the correlation coefficients between each dimension and the correlation coefficients between each dimension and its sub-scale for comparison. The correlation coefficients between each dimension and its sub-scale were higher than those between other dimensions, representing good discriminant validity among all the dimensions.

**Table 1:** Reliability and validity.

**Dimension**	**WAC**	**ST**	**PG**	**CM**	**SS**	**IST**	**RSTR**	**RSTP**	**NE**	**PE**
WAC	**0.735**									
ST	0.610	**0.872**								
PG	0.513	0.589	**0.767**							
CM	0.469	0.611	0.587	**0.818**						
SS	0.527	0.666	0.568	0.634	**0.862**					
IST	0.380	0.541	0.454	0.436	0.418	**0.808**				
RSTR	0.534	0.643	0.546	0.741	0.721	0.418	**0.839**			
RSTP	0.509	0.589	0.570	0.553	0.552	0.588	0.542	**0.846**		
NE	0.415	0.453	0.393	0.392	0.479	0.270	0.399	0.404	**0.881**	
PE	0.408	0.511	0.478	0.487	0.446	0.532	0.453	0.586	0.334	**0.741**
CR	0.680	0.793	0.725	0.803	0.834	0.733	0.782	0.740	0.839	0.788
AVE	0.416	0.434	0.468	0.506	0.503	0.480	0.545	0.489	0.511	0.483

The diagonal bold is the Pearson correlation between dimensions and the sub-scale to which they belong, and the lower triangle is the Pearson correlation between dimensions.

WAC, Weak Attentional Control; ST, Spontaneous Thinking; PG, Psychological Gap; CM, Competition Mood; SS, Somatic Sensation; IST, Irrelevant to the Sports Task; RSTR, Relevant to the Sports Task Result; RSTP, Relevant to the Sports Task Process; NE, Negative Effect; PE, Positive Effect.

### Correlation among MW frequency, content, and bidirectional effects

The correlation analysis showed significant positive correlations among MW frequency, content, and positive and negative effects, as presented in Table 2.

**Table 2:** Descriptive statistics and correlation analysis results of frequency, content, and bidirectional effects of MW.

**Variable**	**M ±SD**	**1**	**2**	**3**	**4**	**5**	**6**	**7**	**8**	**9**
1. FMW	45.025 ± 12.248	1								
2. IST	5.717 ± 2.202	0.549^**^	1							
3. RSTR	6.83 ± 2.157	0.502^**^	0.487^**^	1						
4. RSTP	6.522 ± 2.412	0.678^**^	0.588^**^	0.539^**^	1					
5. NE	13.909 ± 4.217	0.526^**^	0.270^**^	0.374^**^	0.404^**^	1				
6. PE	8.805 ± 2.974	0.571^**^	0.532^**^	0.406^**^	0.586^**^	0.334^**^	1			
7. Gender	–	0.065	−0.003	−0.051	0.059	0.016	0.008	1		
8. Age	20.45 ± 2.407	0.095^**^	0.058	0.118^*^	0.108^**^	0.048	0.052	0.055	1	
9. YT	5.88 ± 3.815	−0.095^**^	−0.108^**^	−0.043	−0.086^*^	0.015	−0.140^**^	0.058	0.302^**^	1

^*^p < 0.05.

^**^p < 0.01.

FMW, Frequency of Mind Wandering; IST, Irrelevant to the Sports Task; RSTR, Relevant to the Sports Task Result; RSTP, Relevant to the Sports Task Process; NE, Negative Effect; PE, Positive Effect; YT, Years of Training.

In addition, the age of the athletes exhibited significant positive correlations with FMW, RSTR, and RSTP. The years of training of the athletes presented significant correlations with all the variables except for RSTR and NE. Therefore, the age and years of training of the athletes were analyzed as control variables in the subsequent analysis.

### Testing the mediating model of different MW contents

On the basis of the correlation analysis, the mediating role of different MW contents in MW frequency and its negative and positive effects was further examined by constructing structural equation models. We generally used the target coefficient (TC) to determine whether the second-order CFA can replace the first-order CFA. The calculation formula is as follows: TC = first-order CFA fully correlated χ^2^/second-order CFA χ^2^. The closer TC is to 1, the more the second-order model can represent the first-order model appropriately, and reaching 0.74 is generally considered acceptable (Doll et al., 1994). In the present study, the frequency of MW was measured as a second-order model, TC = 649.641/687.438 = 0.945, which reached a criterion of 0.74, indicating that the second-order CFA model of the athletes' MW frequency can replace the first-order CFA model.

First, a structural equation model was constructed to examine the direct influence of MW frequency on the negative effect. The goodness-of-fit was acceptable, with indices of χ^2^*/df* = 3.140, root-mean-square error of approximation (RMSEA) = 0.050, standardized root-mean-square residual (SRMR) = 0.040, comparative fit index (CFI) = 0.925, and Tucker–Lewis index (TLI) = 0.916. Among them, the recommended value of model fit, χ^2^*/df* , is as small as possible, which is within 5 in accordance with the wider standard identified by Schumacker and Lomax (2004). The results showed that the direct influence of MW frequency on the negative effect was significant (β = 0.674, *p* < 0.001). Then, the direct influence of MW frequency on the positive effect was examined. Similarly, the goodness-of-fit was acceptable, with indices of χ^2^*/df* = 3.172, RMSEA = 0.051, SRMR = 0.042, CFI = 0.922, and TLI = 0.914. The results indicated that the direct influence of MW frequency on the positive effect was significant (β = 0.60, *p* < 0.001).

**Figure 2:**
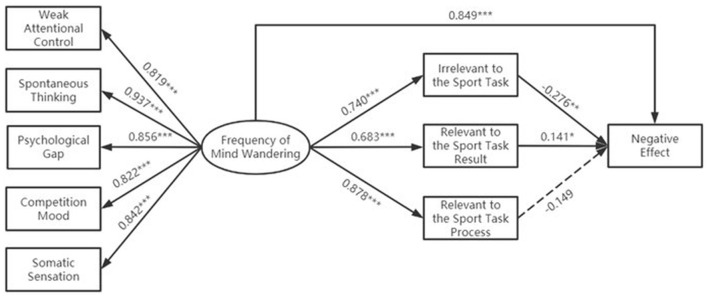
Relationship between the frequency of mind wandering, content of mind wandering, and its negative affect.

Then, a mediation test was conducted for negative effects. The goodness-of-fit was acceptable, with indices of χ^2^*/df* = 2.794, RMSEA = 0.046, SRMR = 0.044, CFI = 0.911, and TLI = 0.903. The results are presented in Figure 2, where the direct influence of the frequency of athletes' MW on the negative effect was significant (β = 0.849, *p* < 0.001). That is, the frequency of MW still positively predicted its negative effect. The frequency of athletes' MW exerted a significant predictive effect on MW contents that were irrelevant to the sports task (β = 0.740, *p* < 0.001), relevant to the sports task result (β = 0.683, *p* < 0.001), and relevant to the sports task process (β = 0.878, *p* < 0.001). MW contents that were irrelevant to the sports task (β = −0.276, *p* < 0.01) and relevant to the sports task result (β = 0.141, *p* < 0.05) were significant predictors of MW negative effect.

**Figure 3:**
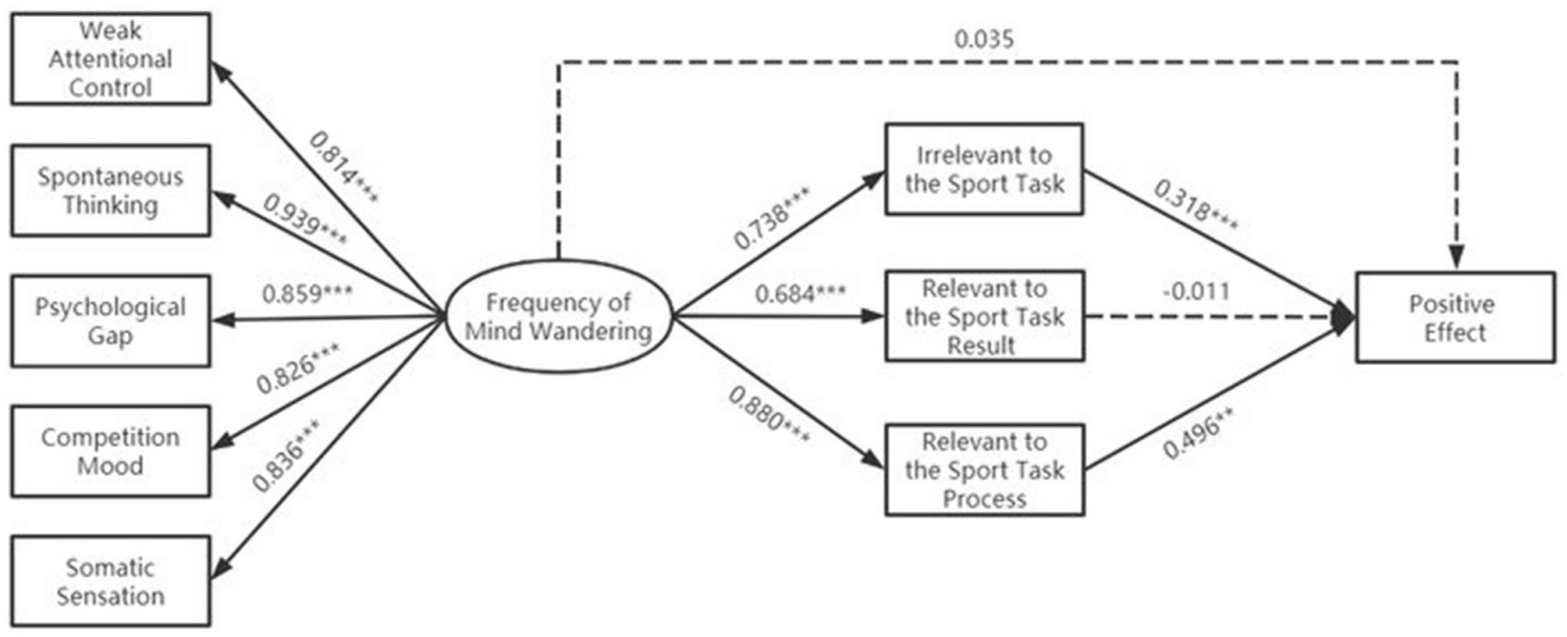
Relationship between the frequency of mind wandering, content of mind wandering, and its positive affect.

Lastly, a mediation test was conducted for positive effects. The goodness-of-fit index was acceptable, with χ^2^*/df* = 2.789, RMSEA = 0.046, SRMR = 0.042, CFI = 0.913, and TLI = 0.905. The results are presented in Figure 3, where the direct influence of the frequency of athletes' MW on positive effect was insignificant (β = 0.035, *p* = 0.821). The frequency of athletes' MW significantly predicted the MW contents that were irrelevant to the sports task (β = 0.738, *p* < 0.001), relevant to the sports task result (β = 0.684, *p* < 0.001), and relevant to the sports task process (β = 0.880, *p* < 0.001). MW contents that were irrelevant to the sports task (β = 0.318, *p* < 0.001) and relevant to the sports task process (β = 0.496, *p* < 0.01) were significant predictors of MW positive effect.

### Indirect effect path test

The results of the mediating effect were analyzed by examining the three different MW contents and comparing the results of the three mediating effects. First, the multiple mediators of negative effect were examined, as indicated in Table 3. The frequency of athletes' MW exerted a significant indirect influence on negative effects through MW contents that were irrelevant to the sports task [β = −0.342, BC 95% confidence interval [CI] = [−0.627, −0.145], percentile 95% CI = [−0.637, −0.149]] and relevant to the sports task result [β = 0.162, BC 95% CI = [0.012, 0.325], percentile 95% CI = [0.022, 0.346]]. Further comparison of the mediating effects revealed that the negative effect from MW contents that were irrelevant to the sports task was significantly smaller than that from MW contents that were relevant to the sports task results [β = −0.504, BC 95% CI = (−0.817, −0.22), percentile 95% CI = (−0.871, −0.266)].

**Table 3:** Comparative analysis of specific indirect effect (negative).

		**Product of coefficient**			**Bootstrap 1,000 times 95% CI**			
	**Point estimate**				**Bias corrected**		**Percentile**	
		**S.E**.	**Est./S.E**.	***P*-value**	**Lower**	**Upper**	**Lower**	**Upper**
**Indirect effect**								
IST	−0.342	0.120	−2.854	0.004	−0.627	−0.145	−0.637	−0.149
RSTR	0.162	0.085	1.918	0.055	0.012	0.325	0.022	0.346
RSTP	−0.220	0.234	−0.940	0.347	−0.734	0.164	−0.728	0.178
Total	1.026	0.095	10.848	0.000	0.843	1.215	0.854	1.233
**Contrasts**								
IST vs. RSTR	−0.504	0.150	−3.363	0.001	−0.817	−0.22	−0.871	−0.266
IST vs. RSTP	−0.122	0.268	−0.456	0.649	−0.616	0.423	−0.627	0.388
RSTR vs. RSTP	0.382	0.259	1.475	0.140	−0.084	0.912	−0.058	0.937

IST, Irrelevant to the Sports Task; RSTR, Relevant to the Sports Task Result; RSTP, Relevant to the Sports Task Process.

Then, the multiple mediators of positive effect were tested for three different MW contents. The results of the comparative analysis of the three mediating effects are provided in Table 4. The results indicated that the MW frequency of the athletes exerted a significant indirect influence on positive effect through MW contents that were irrelevant to the sports task [β = 0.341, BC 95% CI = [0.198, 0.536], percentile 95% CI = [0.198, 0.538]] and relevant to the sports task process [β = 0.635, BC 95% CI = [0.274, 1.093], percentile 95% CI = [0.289, 1.148]]. Further comparison of the results of the mediating effect showed that the positive effect from MW contents that were irrelevant to the sports task was not significantly different from that of the MW contents that were relevant to the sports task process.

**Table 4:** Comparative analysis of specific indirect effect (positive).

		**Product of coefficient**			**Bootstrap 1,000 times 95% CI**			
	**Point estimate**				**Bias corrected**		**Percentile**	
		**S.E**.	**Est./S.E**.	***P*-value**	**Lower**	**Upper**	**Lower**	**Upper**
**Indirect effect**								
IST	0.341	0.086	3.952	0.000	0.198	0.536	0.198	0.538
RSTR	−0.011	0.064	−0.175	0.861	−0.119	0.136	−0.130	0.127
RSTP	0.635	0.219	2.901	0.004	0.274	1.093	0.289	1.148
Total	1.016	0.094	10.812	0.000	0.852	1.213	0.855	1.226
**Contrast**								
IST vs. RSTR	0.352	0.116	3.049	0.002	0.133	0.608	0.130	0.598
IST vs. RSTP	−0.294	0.226	−1.301	0.193	−0.773	0.107	−0.802	0.103
RSTR VS. RSTP	−0.646	0.242	−2.671	0.008	−1.193	−0.215	−1.201	−0.243

IST, Irrelevant to the Sports Task; RSTR, Relevant to the Sports Task Result; RSTP, Relevant to the Sports Task Process.

## Discussion

The present study examined the bidirectional effects of the frequency of athletes' MW and the role of different MW contents through multiple mediation model testing. The frequency of athletes' MW not only directly predicted negative and positive effects but also indirectly predicted negative and positive effects through different MW contents.

### The occurrence of MW in athletes may have bidirectional effects

The occurrence of MW may have a bidirectional effect in training and competition situations through retrospective surveys. First, this study showed that the frequency of MW among athletes positively predicted the negative effect, supporting Hypothesis 1. The result of this study is consistent with previous studies. One study concluded that MW happens when athletes perform sports tasks during training and competition, which leads to negative effects (Latinjak, 2018). Therefore, it is clear that the negative effect of MW on athletes cannot be ignored.

The negative effect of MW on athletes can be interpreted by combining the “decoupling hypothesis” and the “context regulation hypothesis.” The decoupling hypothesis suggests that MW occurs because attention is coupled with internal processing while being decoupled from task-related information (Smallwood et al., 2003; Smallwood and Schooler, 2006; Smallwood, 2010). Athlete's attention is required to focus on tasks, whether in training or competition situations. On the basis of this theory, MW occurs when an athlete's attention is coupled with his/her own internal thoughts and disengaged from the current sports task, resulting in a negative effect on the athlete. Moreover, in accordance with the “context regulation hypothesis,” the task context should be considered when evaluating the effect of MW (Smallwood and Andrews-Hanna, 2013). Individuals who experience MW during sustained attention tasks can exhibit impaired performance (Gouraud et al., 2018). Similarly, the occurrence of MW is detrimental in the context of a sustained attention task, such as training and competition situations, wherein athletes frequently need to process multiple pieces of information. One study also found that MW exerted a negative effect on fine motor movement control, supporting the idea that a decoupling of sensory-motor processes occurs during MW (Dias Da Silva and Postma, 2022).

The present study also showed that MW frequency in athletes positively predicted a positive effect, supporting Hypothesis 1. The result of this study is consistent with previous studies. MW in athletes has positive effects, such as fatigue reduction and mood improvement (Latinjak, 2018). In addition, previous studies have found that MW has positive effects on problem-solving and enhancing individual creativity (Preiss, 2022; Teng and Lien, 2022; Yang and Wu, 2022). MW can be viewed from a switching perspective (Wong et al., 2023). MW among athletes is the switch between two states (from focusing on oneself to the task of the sports) that can facilitate their creative thinking and decision-making, i.e., producing positive effects. Meanwhile, studies have shown that increasing perceptual load may impair, rather than improve, sports performance. Increased task demands can be counterproductive and even extremely dangerous in some situations (Aitken et al., 2023). Sustained cognitive tasks in the sports context will gradually increase an athlete's cognitive load and may exert an opposite effect if the athlete is required to focus on the sports task all the time. In addition, focusing on something other than running itself will improve, rather than impair, running economy during running exercise (Schücker et al., 2009). The “opportunity cost model” (Kurzban et al., 2013) is an important perspective that proposes a role for MW in the experience of boredom and the negative effect that often occurs during ongoing task performance. The model assumes that when the opportunity cost of a low-reward task is too high, our brains have an inherent tendency to seek rewards and stimulation elsewhere. This tendency drives us to engage in other mental tasks that may contribute to greater subjective rewards, including MW. The growing opportunity cost manifests itself in distasteful subjective mental states, including boredom, effort, stress, and fatigue. However, athletes are often in these objectionable subjective mental states during training and competition. At this point, MW plays a positive role in the experience of boredom and negative effects that commonly occur during training and competition. Therefore, MW is not detrimental in all sports situations; that is, MW during training and competition can also have positive effects.

In conclusion, athletes believe that MW can have both positive and negative effects on them. “MW in sporting performance (MWSP)” is a theory of MW that has been proposed specifically in the sports context. MWSP explored the reason for these bidirectional effects. MWSP considers that MW occupies limited attentional resources. The amount of attentional resources occupied by MW varies by its different content, resulting in different attentional resources left for sports tasks; When the attentional resources for sports tasks are reduced, the resources are not able to sustain the task during the competition, and performance will decrease (or vice versa; Li et al., 2024). The “decoupling hypothesis” emphasizes that MW takes up attentional resources, and MWSP builds on this by further suggesting that there are differences in the attentional resources taken up by MW due to its different contents, which ultimately leads to the fact that MW will have different effects on athletes. The present study suggests that we should give considerable attention to the serious negative effect of MW, which may lead to mistakes and missed medals for athletes during competition. Simultaneously, we should not blindly intervene in the occurrence of MW to avoid hiding its positive effect.

### The role of different MW contents

Based on data from the athlete's self-report, the indirect effect test revealed that three different MW contents played different roles in the frequency of MW and its negative effects. Specifically, the frequency of athletes' MW exerted a negative indirect influence on negative effects through MW contents that were irrelevant to the sports task. The frequency of athletes' MW positively influenced negative effects through MW contents that were relevant to the sports task result. Further comparison of the indirect effect results revealed that negative effects from MW contents that were irrelevant to the sports task were significantly smaller than those from MW contents that were relevant to the sports task result. Therefore, the indirect effects differ significantly, supporting Hypothesis 2.

MW contents relevant to the sports task result included rewards and honors, the final outcome of the competition, and the direct result of the motor (Li and Yao, 2016). The negative effect of MW contents that were relevant to the sports task result may be due to the fact that athletes recognize the importance of the competition and focus excessively on the rewards. However, it does not help athletes improve or maintain their performance; rather, it has the opposite effect. This type of additional attention is generally associated with the conscious control of the motor process, which can disrupt the automatic execution of the original action, leading to changes in movements that athletes are already accustomed to Belletier et al. (2015). Then, it produces a detrimental effect. In addition, the indirect effect of MW contents, which was irrelevant to the sports task, was significant but had a negative effect. MW contents that are irrelevant to the sports task included post-competition activities, life events, relatives, and friends (Li and Yao, 2016). Athletes may wonder about the expectations of their relatives and friends during training and competition. These expectations will be converted into motivation for athletes to improve their sports performance. Relevant evidence was provided by one study that motor learning benefits when expectations are enhanced (Simmonds et al., 2023). Therefore, MW contents that are irrelevant to the sports task may function as motivators for athletes, which explains the result that it is a negative predictor of negative affect in another way. Finally, the indirect effect of MW contents that were relevant to the sports task process was not significant. The reason for this result may be that the athlete's attention was not disengaged from the sports task while he or she was thinking about the content related to the sports task. The consistency of attention to the sports task protected it from the negative effects.

Meanwhile, based on athlete self-reported data, the present study found that three different MW contents play different roles in the frequency of MW and its positive effects. Specifically, the frequency of MW in athletes exerted a positive indirect influence on positive effects through MW contents that were irrelevant to the sports task and relevant to the sports task process. Further comparison of the indirect effects showed that the differences were insignificant, partially supporting Hypothesis 3. MW frequency in athletes had a positive effect through MW content that was irrelevant to the sports task. This result may be due to the training and competition contexts being continuous cognitive and physical activities under high pressure, gradually depleting the cognitive resources and physical exertion of athletes over time and causing them to feel fatigued. The occurrence of MW allows athletes to temporarily disconnect from their stressful environment and let their minds rest and relax appropriately to relieve fatigue. This situation is similar to the previous finding in which MW appears to promote a positive mood during a runner's training (Miś and Kowalczyk, 2021). In addition, we found an indirect positive effect of MW content that is relevant to the sports task process. This type of MW content will change with the course of a game and includes the next segment of the competition, contextual change, opponent-related, and action-related (Li and Yao, 2016). On the basis of the “current concerns hypothesis,” an individual's MW content is associated with personal goals or uncompleted tasks, and it will facilitate anticipation and planning for future goals. This result is similar to previous research findings, in which MW can lead to problem-solving and planning for personal, relevant future goals. The prospective nature of MW may be functional; that is, future-oriented MW allows people to plan and think about their future goals (Mooneyham and Schooler, 2013). In the sports context, information related to the sports task process constitutes the current focus of athletes. To win the game, athletes may plan and organize the entire sports process. The content of these thoughts is also part of the athlete's personal relevant goals. The occurrence of MW in athletes is the process of approaching personal goals, which is beneficial for athletes' performance in the sports process and, thus, exerts a positive effect. However, we did not find a significant indirect effect of MW contents relevant to the task of the sports, resulting in a positive effect. As discussed above, excessive focus on the result by athletes can lead to increased stress, disruption of the original, inherent automatic procedures of movements, and consequently, the production of negative effects, such as motor errors. Therefore, coaches and athletes can use MW content and try to guide the positive effect it brings to training and competition. These results are in accordance with the results of the above negative model, and they also validate the accuracy of the present study.

In summary, athletes believe that the impact of MW will vary depending on its content. The findings suggest that the different contents of MW need to be taken into account when studying the effects of MW on athletes in future research. Through athletes' self-reports, the current study not only analyzed the relationship between different MW contents and negative effects but also the relationship with positive effects. The present study shows that the results in the two opposite directions are not contradictory and serve to validate each other. Our survey results support the “content regulation hypothesis” that MW exerts different effects depending on the content features. It is worthwhile to recognize that this questionnaire-based study was conducted with athletes in training and competition situations. The findings enrich the research on the effects of MW in different contexts. The findings suggest that coaches attempt to guide the thinking habits of athletes in sports practice. In particular, less attention should be given to the results and more to the process. Allowing athletes to think about content unrelated to the sports task at the interval of the competition and training can play a positive role in relieving mood and fatigue. Hence, athletes believe that not all MW is harmful, and blindly reducing MW is unscientific. Before measuring its pros and cons, we must consider the specific content features of MW.

### Limitations and future directions

However, this study has some limitations that require improvement in future studies. First, this study focused on MW and its effects in the competitive sports context without distinguishing in detail between training and competition. It also did not distinguish among the characteristics of different sports. To make the findings more targeted to guide practice, future research can refine the occurrence of the MW context (training or competition) in specific sports. Second, the present study classified the content of athletes' MW in relation to sports tasks. This classification is based on preliminary qualitative research, and all the factors have achieved satisfactory validity test results. Future research can select athletes from other countries and regions to verify the feasibility of this classification further. In addition, future researchers are encouraged to classify MW content from other perspectives, such as affective valence and time orientation. Third, athletes' MW in this study was collected through a retrospective questionnaire. Compared with the method of online probes for athletes' inner experience, a retrospective questionnaire is easy to operate and does not destroy the natural state of thinking. However, different methods have their merits and limitations, and a retrospective questionnaire is subjective and cannot verify causality between variables. Future research can consider different approaches, e.g., using online probes in a laboratory or a simulated competition context to collect data and enhance the reliability of the data analysis results. In particular, we suggest that future experimental studies can build on the results of the present study to further validate the causal relationship between different MW contents and athletic performance in sports.

## Conclusion

Based on data from a questionnaire survey of athletes, this study used structural equation models to examine the bidirectional effects of MW in sports and the role of different MW contents. The frequency of MW can positively predict its bidirectional effects. MW content plays a multiple mediating role between them, but the direction of influence varies with different contents. The results highlight the importance of MW content. In particular, athletes' MW exerts the strongest negative effect through contents relevant to the sports task result. It has an equally positive effect through content that is irrelevant to the sports task and relevant to the sports task process. The results suggest that managing MW content is a promising intervention method. It has important theoretical and practical implications for improving sports performance.

## Data availability statement

The raw data supporting the conclusions of this article will be made available by the authors, without undue reservation.

## Ethics statement

The studies involving humans were approved by Ethics Committee: Heibei Normal University; No. 2023LLSC031. The studies were conducted in accordance with the local legislation and institutional requirements. The participants provided their written informed consent to participate in this study.

## Author contributions

JL: Conceptualization, Funding acquisition, Project administration, Supervision, Writing – original draft. CL: Methodology, Writing – review & editing. SX: Data curation, Writing – review & editing. YH: Conceptualization, Supervision, Writing – original draft.

## Footnotes

^1^ http://sports.163.com/11/0224/09/6TL9TB7T00051CDG.html

^2^ https://www.sohu.com/a/497762179121048740
